# Prognostic significance of PD‐L1 expression on cell‐surface vimentin‐positive circulating tumor cells in gastric cancer patients

**DOI:** 10.1002/1878-0261.12643

**Published:** 2020-02-28

**Authors:** Mengyuan Liu, Ruoyu Wang, Xuren Sun, Yuting Liu, Zhi Wang, Jin Yan, Xiangyu Kong, Shanshan Liang, Qiuge Liu, Tong Zhao, Xuening Ji, Gang Wang, Fuguang Wang, Guan Wang, Liang Chen, Qingfu Zhang, Weipeng Lv, Heming Li, Mingjun Sun

**Affiliations:** ^1^ Department of Gastroenterology The First Affiliated Hospital of China Medical University Shenyang China; ^2^ Department of Endoscopy The First Affiliated Hospital of China Medical University Shenyang China; ^3^ Department of Oncology Affiliated Zhongshan Hospital of Dalian University China; ^4^ The Key Laboratory of Biomarker High Throughput Screening and Target Translation of Breast and Gastrointestinal Tumor Dalian China; ^5^ Department of Gastrointestinal Surgery Affiliated Zhongshan Hospital of Dalian University China; ^6^ Department of Radiology The First Affiliated Hospital of China Medical University Shenyang China; ^7^ Department of Computer Science College of Engineering Shantou University China; ^8^ Key Laboratory of Intelligent Manufacturing Technology of Ministry of Education Shantou University China; ^9^ Department of Pathology The First Affiliated Hospital and College of Basic Medical Sciences of China Medical University Shenyang China; ^10^ Department of Pathology Affiliated Zhongshan Hospital of Dalian University China

**Keywords:** cell‐surface vimentin, circulating tumor cells, epithelial–mesenchymal transition, gastric cancer, programmed cell death ligand 1

## Abstract

Although circulating tumor cells (CTCs) have shown promise as potential biomarkers for diagnostic and prognostic assessment in gastric cancer (GC), determining the predictive and prognostic value of programmed death‐ligand 1 (PD‐L1)‐positive CTCs in patients with GC is a challenge. Here, we identified that the expression of total vimentin (VIM) protein was positively correlated with PD‐L1 and inhibited CD8^+^ T‐cell activation in patients with GC according to bioinformatics analysis. Notably, coexpression of PD‐L1 and cell‐surface VIM (CSV) was detected by immunofluorescence and immunohistochemistry assay in locally advanced GC tumor specimens and metastatic lymph nodes. Likewise, CSV expression level was significantly decreased after transiently knocking down PD‐L1 in GC cell lines. Based on our established CTC detection platform, CTCs were isolated from peripheral blood samples collected from 70 patients (38 resectable and 32 unresectable) with GC using magnetic positive selection and a CSV‐specific monoclonal antibody, 84‐1. CSV^+^PD‐L1^+^CTCs were observed in 50 of 70 (71%) GC patient samples, ranging from 0 to 261 mL^−1^. A higher number of CSV^+^PD‐L1^+^CTCs were significantly associated with a short survival duration and poor therapeutic response. This study demonstrated that detection of PD‐L1^+^CTCs using a CSV‐enrichment method has promising value as a clinically relevant prognostic marker for GC.

## Graphical abstract

CSV^+^PD‐L1^+^CTC exhibits epithelial mesenchymal transition subtype, which can be observed in GC patient’s peripheral blood samples. A higher number of CSV^+^PD‐L1^+^CTCs are significantly associated with a short survival duration and poor therapeutic response.

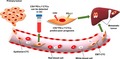

**Abbreviations**
CIs: confidence intervals
CSV: cell‐surface vimentin
CTCs: circulating tumor cells
EMT: epithelial–mesenchymal transition
EpCAM: epithelial cell adhesion molecule
GC: gastric cancer
HER‐2: human epidermal growth factor receptor 2
HR: hazard ratios
IHC: immunohistochemistry
OS: overall survival
PBMCs: peripheral blood mononuclear cells
PD‐L1: programmed death‐ligand 1
PFS: progression‐free survival
RECIST: Response Evaluation Criteria in Solid Tumors
ROC: receiver operating characteristic curve

## 1 Introduction

Gastric cancer (GC) represents the fifth most common malignancy and the second leading cause of annual cancer‐related deaths worldwide, including Asia (Lyons *et al.*, 2019; Segal *et al.*, 2018). Although patients have shown an impressive improvement in response to radio‐, chemo‐, and molecular‐targeted therapies, those with locally advanced or metastatic GC continue to have a poor prognosis: the 5‐year survival rate for advanced disease is still < 20% (Chen *et al.*, 2016; Segal *et al.*, 2018). Thus, improved outcomes for advanced GC by the early selection of patients with a potential risk of recurrence are urgently needed.

Circulating tumor cells (CTCs) are cell populations that act as cancer seeds by detaching from primary lesions, entering blood vessels and thus causing metastasis (Hamilton and Rath, 2018). Increasing evidence has demonstrated that the cellular process in which epithelial cells acquire a mesenchymal phenotype (epithelial–mesenchymal transition, EMT) leads to an increase in cellular invasion and spread (Diepenbruck and Christofori, 2016; Dongre and Weinberg, 2018). Such EMT–CTC subpopulations are considered the roots of metastases (Kölbl *et al.*, 2016). Currently, selecting specific markers for the detection of EMT–CTCs is an enormous challenge facing those in the CTC technology field (Yahyazadeh Mashhadi *et al.*, 2019). Cytoplasmic vimentin (VIM) is overexpressed and transports to the cell surface during the EMT process (Satelli and Li, 2011; Satelli *et al.*, 2014). Hence, cell‐surface VIM (CSV) could be involved in tracking CTCs to a metastatic site and assisting circulating cells to reseed the metastatic niche; enrichment of CTCs by CSV allows for detection of cells with mesenchymal features that might be missed by conventional epithelial cell adhesion molecule (EpCAM)‐dependent methods. A recent study performed in our laboratory has further demonstrated that the detection of CSV^+^CTC parallels the therapeutic response and the prognosis in patients with advanced GC based on a CSV‐specific mAb, 84‐1.

However, as a heterogeneous cell population, some CTCs manage to evade the immune system of the host and exposure to immune‐mediated destruction (Smith and Kang, 2013). It has been demonstrated that antitumor therapy, including radiation and chemotherapy, may induce immune activation and cytokine secretion in the tumor microenvironment (Kaur and Asea, 2012; Kershaw *et al.*, 2013). The tumor immune status may be involved in the response to different therapeutic strategies. Therefore, a better understanding of the characteristics of CTCs and their crosstalk with immune cells may shed light on potential opportunities of therapeutic response prediction in GC. Nowadays, checkpoint pathway blockade of programmed cell death ligand 1 (PD‐L1) is a highly promising immunotherapy in a subset of patients with a broad spectrum of cancers that involves activating T lymphocytes and enhancing antitumor immunity (Zou *et al.*, 2016). In line with this, analysis of the PD‐L1 expression level in CTCs is currently in the exploratory stage. Herein, in this present study, we sought to evaluate PD‐L1 expression on CSV^+^CTCs in a cohort of resectable and unresectable patients with GC receiving treatment at our institution in order to provide new insights into the development of a therapy for GC.

## 2 Materials and methods

### 2.1 Validation of the expression of vimentin in GC

Oncomine (https://www.oncomine.org/) and Gene Expression Profiling Interactive Analysis (GEPIA, http://gepia.cancer-pku.cn/) were applied to verify VIM expression in GC (Rhodes *et al.*, 2007; Tang *et al.*, 2017). The prognostic value of VIM was determined by Kaplan–Meier analysis using KM plotter online software (http://kmplot.com/analysis/) on 1065 patients with GC (2017 version) (Szász *et al.*, 2016). The VIM gene (probe set, 201426_s_at) was entered into the KM plotter database (http://kmplot.com/gastric/) to obtain a Kaplan–Meier survival plot. Hazard ratios (HR) with 95% confidence intervals (CIs) and log‐rank *P*‐values were calculated and displayed on the webpage. The endpoints of interest were progression‐free survival (PFS) and overall survival (OS).

### 2.2 Potential functions and pathways associated with vimentin

To further investigate potential functions and pathways associated with VIM, the Gene Set Enrichment Analysis (GSEA) was performed using R package ‘fgsea’ from Bioconductor in http://www.ncbi.nlm.nih.gov/geo/query/acc.cgi?acc=GSE62254 (Subramanian *et al.*, 2005). Parameters used for the analysis were as follows. The Gene sets of cancer hallmarks from MSigDB were used for running GSEA and 1000 permutations were used to calculate the *P* value. The *P* < 0 .01 was used to select statistically significant gene sets.

### 2.3 Analysis of immune cells composition from gene expression data and evaluation of the correlation of vimentin and PD‐L1

Gene Expression Omnibus (GEO) 15459, 62254, and The Cancer Genome Atlas (TCGA)‐STAD datasets were split into quartiles according to VIM expression. We used the online analytical platform CIBERSORT (https://cibersort.stanford.edu/) in order to estimate the relative proportions of 22 immune cell types (Newman *et al.*, 2015). Analyses were performed with 100 permutations, and enabled quantile normalization and default statistical parameters. We used r 3.5.3 for statistical analysis and graphpad prism 7 (GraphPad Software, San Diego, CA, USA) for data presentation. Student's *t*‐test analysis was used to assess the CD8 T cell between low‐ and high‐VIM groups. The gene expression values of VIM and PD‐L1 were median‐centered. Spearman’s rank correlation analysis was used to assess the relationship between these in each dataset.

### 2.4 GSVA and calculation of EMT score

Gene Set Variation Analysis (GSVA) (Hanzelmann *et al.*, 2013) is a nonparametric, unsupervised method for estimating variation of gene set enrichment through the samples of an expression data set and bypasses the conventional approach of explicitly modeling phenotypes within the enrichment scoring algorithm. To infer specific activated pathways related with PD‐L1 expression, GSVA was performed to calculate the enrichment score of mesenchymal–phenotype gene signatures for each patient in TCGA GC datasets. The signatures whose name including ‘Mes‐phenotype’ were selected and applied to the correlation tests for the relationship between PD‐L1 levels and the activation scores of the mesenchymal‐related signatures. Mesenchymal–phenotype genes were listed in Table S1.

### 2.5 Patient eligibility and recruitment

Peripheral blood samples from 70 patients diagnosed with resectable and unresectable GC disease were collected at the Zhongshan Affiliated Hospital of Dalian University. Key inclusion criteria for resectable patients were having a plan for (presurgery) or have received a radical gastrectomy and D2 node dissection for GC (postsurgery). Patients with unresectable disease were those with locally advanced tumors that could not be removed by radical surgery or patients with metastatic disease. Patients with infections or secondary malignant tumors were excluded from this study. Clinicopathological information was recorded for each patient after enrollment. Response Evaluation Criteria in Solid Tumors (RECIST) were utilized to access disease status for each patient with advanced GC. Patients were grouped as responding (stable/partial response/complete response) and nonresponding (progression) based on RECIST criteria. This study was approved by the Zhongshan Affiliated Hospital of Dalian University Institutional Review Board (Protocol: 2015‐032). All patients enrolled in this study supplied informed written consent. The study methodologies conformed to the standards set by the Declaration of Helsinki.

### 2.6 Blood collection and processing

A total of 5 mL of peripheral blood was drawn from each enrolled patient before or after intravenous chemotherapy drugs for at least 7 days. Peripheral blood mononuclear cells (PBMCs) were harvested as previously described. Briefly, peripheral blood samples were collected in 10‐mL vacutainer tubes with K2‐EDTA (BD Diagnostic Systems, Franklin Lakes, NJ, USA). Secondly, blood samples were diluted with PBS containing 2% FBS at a 1 : 1 dilution and layered carefully over 3–4 mL of Ficoll‐Paque PLUS density gradient medium (Ficoll‐Paque PREMIUM) in a 15‐mL centrifuge tube (SepMate tube; StemCell Technologies, Vancouver, BC, Canada). PBMCs were harvested by pipetting the top layer and washed twice at RT. All PBMC samples were isolated within 2 h to ensure the best enrichment of CTCs based on our previous publication.

### 2.7 CTC enrichment

Circulating tumor cells were enriched and detected as previously described. Briefly, CD45^+^ cell populations were depleted from PBMCs using an EasySep Human CD45 Depletion Kit (StemCell Technologies) according to the manufacturer’s protocol. CD45^–^ cell populations were subjected to CSV magnetic positive selection using an 84‐1 antibody (a specific marker for CSV) and to EpCAM‐positive selection, followed by mouse IgG‐microbead binding (Miltenyi Biotec, Bergisch Gladbach, Germany). The cells labeled with 84‐1 or EpCAM antibodies were then pulled down using a magnetic column (Miltenyi Biotec). The cells labeled with CSV^+^CD45^−^/ EpCAM^+^CD45^−^ were then ready for further analysis.

### 2.8 Immunofluorescence imaging

The cell pellet extracted above was mixed with MACS buffer (Miltenyi Biotec) and stained with the 84‐1 antibody for 1 h at RT. Then, cells were cytospinned onto microscope adhesion slides (Thermo Fisher Scientific, Waltham, MA, USA) using CytoFuge (Iris International, Chadsworth, CA, USA) followed by blocking with 1% FBS in PBS for 1 h and fixing by 4% paraformaldehyde (Fisher Scientific) for 10 min. For the staining of PD‐L1, CD45, EpCAM and human epidermal growth factor receptor 2 (HER‐2; Cell Signaling Technology, Danvers, MA, USA), selected cells were permeabilized for 15 min followed by incubation with primary antibody overnight at 4° C. Then, the slides were stained with Alexa Fluor‐488 for 84‐1, Alexa Fluor‐555 for other markers, and DAPI for nuclei for 1 h in a dark room at RT. Immunofluorescent images were captured under a 100× oil objective and analyzed by confocal acquisition software fv10‐asw 3.0 (Olympus, Tokyo, Japan).

### 2.9 Immunohistochemistry

The expressions of HER‐2, VIM, and PD‐L1 in GC tissue samples were detected using immunohistochemistry (IHC) staining. Each GC tissue was cut into 3‐mm sections. All sections were sequentially deparaffinized and dehydrated following the protocol of the S‐P immunohistochemical kit (Zhongshan Jinqiao Biological Technology Ltd., Beijing, China). After blocking for 1 h at RT with 10% normal serum (Invitrogen, Carlsbad, CA, USA), the sections were incubated with anti–HER‐2 (Cell Signaling Technology) or antivimentin antibody (Maixin Biological Technology Development Co., Fujian, China) overnight in a moist box at 4 °C. A DAB kit was used for immune complex visualization (Zhongshan Jinqiao Biological Technology Ltd.). The results were examined by at least two independent pathologists. For PD‐L1 staining, slides were processed on the Autostainer Link 48 (Dako AS480, Glostrup Kommune, Denmark) using an automated staining protocol validated for the PD‐L1 IHC assay with anti‐PD‐L1 22C3 primary antibody (Dako). Our Clinical Pathology Laboratory Centre has been approved of PD‐L1‐IHC quality assessment by the Pathology Quality Control Centre (PQCC) of the National Health (Certification number: 2019‐PQCC‐C3). All the slides were evaluated under an inverted TS100 microscope at a ×200 magnification (Nikon Corporation, Tokyo, Japan). The positive IHC result for PD‐L1 was defined as tumor proportion score > 1%.

### 2.10 Cell culture

Human GC cell lines were kindly provided by Y. Liu (China Medical University). All GC cells were grown in RPMI‐1640 medium (Gibco; Thermo Fisher Scientific) containing 10% FBS, penicillin (10 U·mL^−1^) and streptomycin (100 mg·mL^−1^) in a humidified atmosphere of 5% CO_2_ at 37° C. Cells showing a viability > 98% were used for experiments.

### 2.11 Transient PD‐L1 knockdown

Gastric cancer cells were seeded at a density of 2.5 × 10^5^ cells/well in 6‐well plates. The cells were transiently transfected with siRNA specifically against human PD‐L1 using Lipofectamine transfection agent (Ambion; Thermo Fisher Scientific Inc) according to the manufacturer’s instructions. The PD‐L1 siRNA sequence was 5′‐CCAGCACACUGAGAAUCAATT‐3′ (sense), 5′‐UUGAUUCUCAGUGUGCUGGT T‐3′ (antisense). The control sequence was UUCUCCGAACGUGUCACGUTTACG UGACACGUUCGGAGAATT. Western blot analysis was performed to verify gene‐silencing efficiency.

### 2.12 Cell migration and invasion assays

Cell migration was measured using Transwell chambers with 8.0‐µm pore size membranes (BD Biosciences, San Jose, CA, USA). GC cells that were transfected with siRNA (1 × 10^4^ cells/well) were seeded into each upper chamber and inserted into each lower chamber of 24‐well culture dishes containing 500 μL of medium and 2.5% FBS. After 24‐h incubation, the nonmigrated cells in the upper chamber were carefully removed with a cotton swab. Then, the migrated cells on the outer side of the membrane were fixed with 4% formaldehyde for 1–2 min and stained with a 0.1% Giemsa stain solution. The numbers of migrated cells were counted in five different fields and pictured under the microscope at ×10 magnification.

The cell invasion assay was detected using Matrigel invasion chambers (BD Biosciences). A total of 50 µg of Matrigel (BD Biosciences) was used to coat each upper chamber. GC cells that were transfected with siRNA in serum‐free medium were added into each upper chamber. RPMI 1640 supplemented with 10% FBS was added to each lower chamber. After incubation for 48 h at 37 °C, the membrane facing the lower chamber containing invaded cells was gently removed and mounted on a glass slide. The subsequent steps for fixation, staining, and enumeration of cell numbers were described as mentioned above.

### 2.13 Western blot analysis

Gastric cancer cells were seeded at 2 × 10^5^ cells per well in 6‐well plates and incubated overnight. Total protein was extracted using radioimmunoprecipitation assay lysis buffer according to the manufacturer’s instructions (Beyotime Biotechnology, Nanjing, China). Western blotting was undertaken as described in our previous study (Li *et al.*, 2014). GAPDH was tested as a loading control for western blots in the same sample panel, and densitometric results were analyzed with imagej software (Bethesda, MD, USA).

### 2.14 Flow cytometry

A total of 5 × 10^5^ GC cells were detached with EDTA buffer (1 mm) after transiently being transfected with PD‐L1 siRNA for 48 h. For CSV analysis of the cell surface, cells were stained with 84‐1 monoclonal antibody (1 : 100); mouse primary antibody (CST, Danvers, MA, USA) was used as an isotype control. Later, cells were labeled with a secondary antibody using Alexa Fluor‐488 (CST) after washing twice in PBS. These cells were used for data acquisition immediately using an Attune flow cytometer followed by rinsing twice in PBS. The data were further analyzed using flowjo software (TreeStar Inc, Ashland, OR, USA).

### 2.15 Human magnetic Luminex assay (ELISA)

Plasma PD‐L1 was measured using human magnetic Luminex assay according to the manufacturer’s instructions (LXSAHM; R&D Systems, Minneapolis, MN, USA).

### 2.16 Whole‐genome sequencing

Whole‐genome sequencing was performed using NovaSeq platform at Novogene Bioinformatics Technology Co., Ltd. (Beijing, China). Briefly, genomic DNA was extracted following the general protocol for genome sequencing. Preparation of sequencing libraries and DNA capture methods were completed according to the manufacturer’s instructions (Illumina Truseq Library Construction; Illumina, Inc., San Diego, CA, USA). The DNA fragments were then sequenced using the Illumina PE 150 sequencing system. The stomach specific genes were loaded from https://bioinfo.uth.edu/TissGDB/gene (Kim *et al.*, 2018).

### 2.17 Tumor dissociation

The GC tumor tissues and metastatic lymph nodes were dissociated into single‐cell suspensions using Dulbecco’s modified Eagle’s medium (Invitrogen) containing collagenase IV (1000 U·mL^−1^; Sigma, Saint Louis, MO, USA) and DNase I (0.1 mg·mL^−1^; Sigma) immediately after surgical resection. The cell populations were incubated at 37 °C for 1 h with slow shaking. The isolated single cells were harvested into a 50‐mL conical tube after filtering through a 40‐mm nylon cell strainer (BD Biosciences). Total cells were counted after red blood cell lysis and two times washing in PBS containing 2% FBS at room temperature.

### 2.18 Statistical analysis

All the data reported in this study are expressed as the mean ± standard deviation. All statistical tests were performed using a prism software program (GraphPad Software). Differences in baseline characteristics and CTC counts among patients were analyzed using *t*‐tests. Correlation trend was detected using Spearman’s rank correlation coefficient. The optimal threshold of CTC counts was assessed by constructing a receiver operating characteristic curve (ROC). A log‐rank test was used to compare survival curves for individual groups. HR and 95% CIs are presented in the data. *P* values < 0.05 were considered significant.

## 3 Results

### 3.1 Vimentin was upregulated in diffuse GC tissues and associated with a poor prognosis

**Figure 1:**
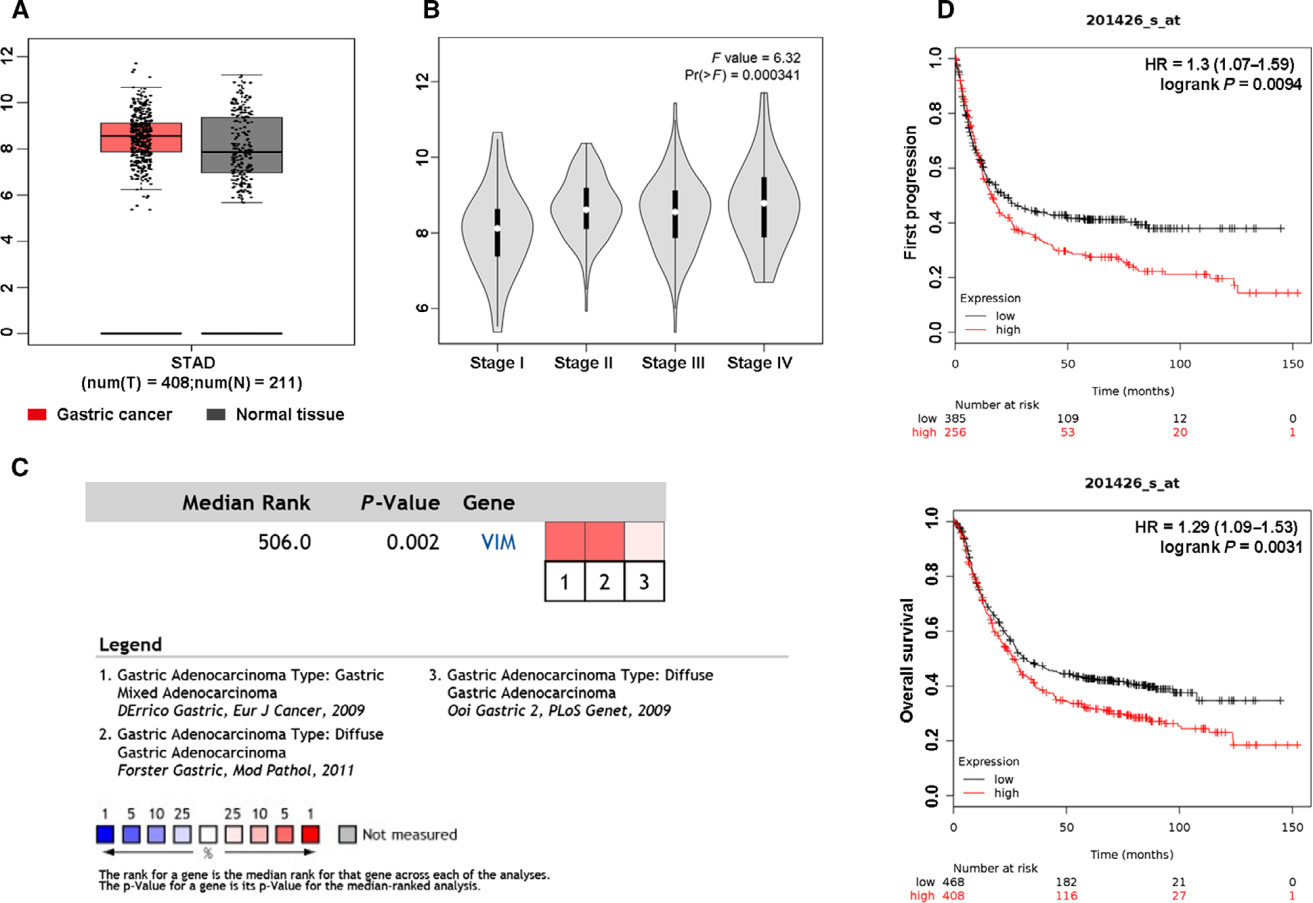
Relationship between VIM expression and clinicopathological features and prognosis. (A) The GEPIA database was used to analyze the expression of VIM in GC tissues (red color) compared to normal tissues (gray color). (B) The GEPIA database was used to analyze the relationship between VIM expression and pathological stages of GC; F value was representative of the *F*‐test. (C) The Oncomine database was used to analyze the expression of VIM in diffuse GC. Meta‐analysis of gene expression profiling for VIM gene in diffuse GC, with *P* < 0.05 and fold change > 1.5. The colored squares indicated the median rank for VIM across each analysis comparing with diffuse GC. (D) First progression survival and OS curves of GC patients with high or low VIM expression using Kaplan–Meier plotter analysis.

Intracellular VIM is a classical EMT marker and translocates on the tumor cell surface during the EMT process in late cancer disease (Satelli *et al.*, 2014). In this study, we assessed the correlation of total VIM expression with the pathological stages of GC using a GEPIA database. We observed that VIM was upregulated in GC tissues compared to corresponding normal tissues (Fig. 1A). Further, higher VIM expression was significantly correlated with pathological stages of GC (*P* < 0.001, Fig. 1B). Next, Oncomine database analysis indicated that increased VIM expression was significantly associated with diffuse GC compared with gastric intestinal type adenocarcinoma based on results from three clinical cohorts (*P* = 0.002, Fig. 1C). Furthermore, we characterized the association between VIM mRNA expression and prognosis in GC using a Kaplan–Meier plotter database. A higher expression level of VIM was significantly associated with shorter first progression survival and OS (Fig. 1D). Accordingly, these results demonstrated that the overexpression of VIM was able to predict a late disease status and poor prognosis in patients with GC.

### 3.2 Relationship of vimentin with immune‐related pathways and PD‐L1 in GC patients

**Figure 2:**
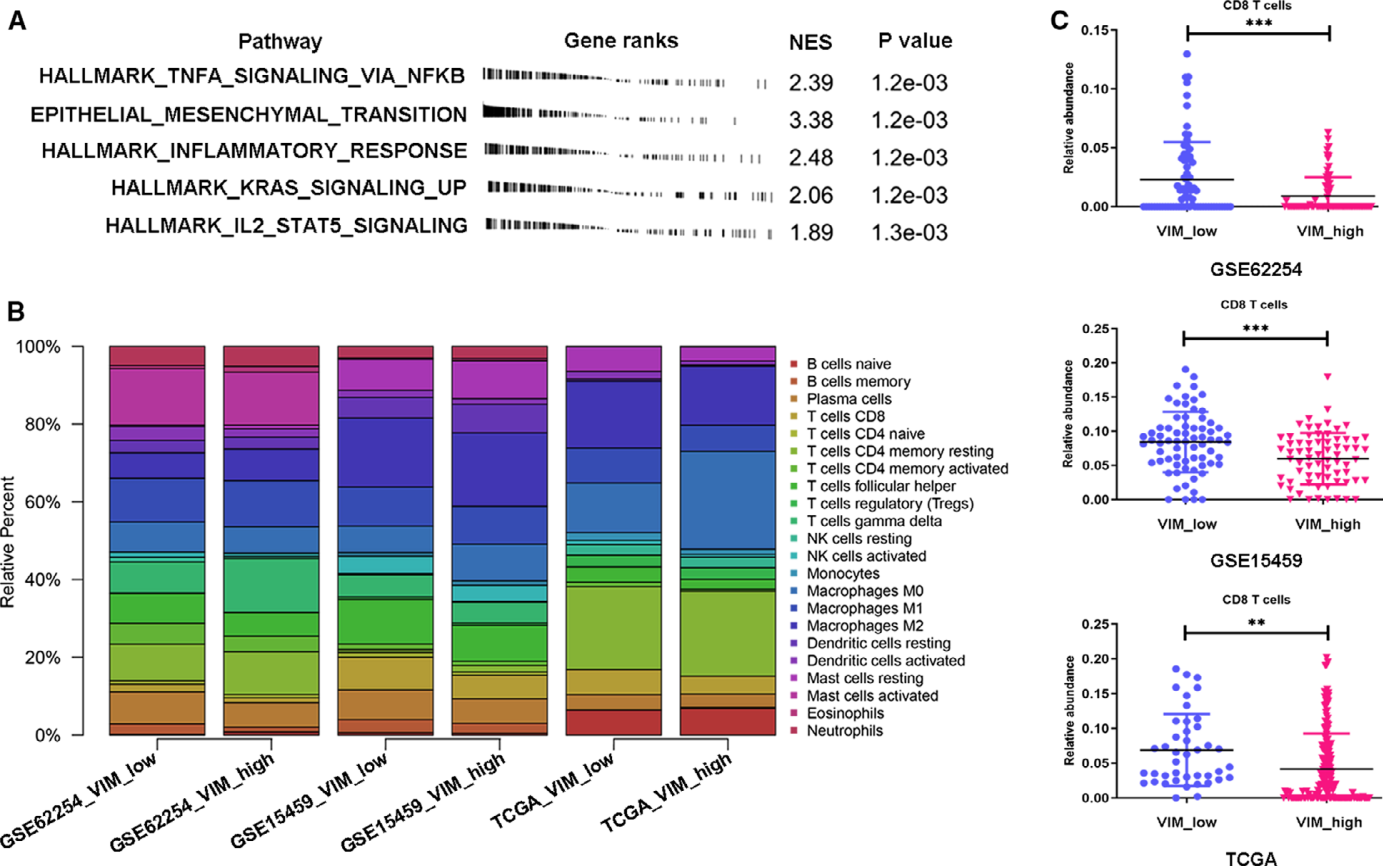
The functional analysis and immune cell fraction with VIM expression across GC patients. (A) GSEA analysis of high expression of VIM‐related genes using the hallmark gene set. The top five most‐enriched pathways are shown. (B) CIBERSORT immune cell fractions were determined for high expression of VIM‐related GC patients in http://www.ncbi.nlm.nih.gov/geo/query/acc.cgi?acc=GSE62254, http://www.ncbi.nlm.nih.gov/geo/query/acc.cgi?acc=GSE15459, and TCGA databases. (C) Mean values and standard deviations for CD8 cells were calculated for low‐ and high‐VIM expression. **P < 0.01; ***P < 0.001.

To further explore VIM‐related biological functions in GC, we performed an unbiased GSEA with respect to the expression of VIM according to the http://www.ncbi.nlm.nih.gov/geo/query/acc.cgi?acc=GSE62254 database using Hallmark Gene Sets from a Molecular Signature Database. We identified that the top five significant pathways were closely related to the indicated immune stimulations, including TNFA signaling and an inflammatory response, IL2‐STAT5 signaling (Fig. 2A). The analysis demonstrated that the level of VIM expression was markedly correlated with immune status in patients with GC.

Next, we used a novel CIBERSORT approach to compare immune cell composition between low/high VIM groups (comparing the upper 25% vs. the lower 25%). A CIBERSORT immune cell profile for each patient was calculated from three separate GC microarray gene expression data (http://www.ncbi.nlm.nih.gov/geo/query/acc.cgi?acc=GSE15459, http://www.ncbi.nlm.nih.gov/geo/query/acc.cgi?acc=GSE62254, and TCGA). These results were shown in Fig. 2B. We also compared mean values for each immune cell type between patients with low and high expression levels of VIM (Table 1). As shown in Table 1, CD8 T cells, plasma cells, follicular helper T cells, and activated dendritic cells were strongly depleted, whereas M0 macrophages were increased in the high VIM expression group. However, the percentage of naïve B cells, memory B cells, CD4 naïve T cells, CD4 memory resting T cells, CD4 memory activated T cells, NK cells, monocytes, M1 and M2 macrophages, dendritic cells, mast cells, eosinophils, and neutrophils remained constant between low/high VIM groups. Through CIBERSORT results analysis, CD8^+^ T‐cell activation was found significantly inhibited in patients with GC showing high VIM expression (Fig. 2C). All of these above results indicated that high VIM expression was related with immune escape in GC.

**Table 1:** Comparison of CIBERSORT immune cell fractions between low‐ and high‐VIM group in http://www.ncbi.nlm.nih.gov/geo/query/acc.cgi?acc=GSE41998, http://www.ncbi.nlm.nih.gov/geo/query/acc.cgi?acc=GSE62254, and TCGA.

	http://www.ncbi.nlm.nih.gov/geo/query/acc.cgi?acc=GSE41998			http://www.ncbi.nlm.nih.gov/geo/query/acc.cgi?acc=GSE62254			TCGA		
	Mean ± SD		*P* values	Mean ± SD		*P* values	Mean ± SD		*P* values
	VIM low	VIM high	VIM high vs. low	VIM low	VIM high	VIM high vs. low	VIM low	VIM high	VIM high vs. low
B cells naive	0.006 ± 0.0157	0.0046 ± 0.0164	0.6157	0.002 ± 0.0055	0.0078 ± 0.0152	**0.0221**	0.0643 ± 0.0383	0.0687 ± 0.0546	0.6745
B cells memory	0.0339 ± 0.0276	0.0258 ± 0.021	0.0540	0.0268 ± 0.0351	0.0129 ± 0.0213	**0.0049**	0.0001 ± 0.0004	0.0029 ± 0.0126	0.1931
Plasma cells	0.0765 ± 0.0252	0.0629 ± 0.0217	**0.0009**	0.0821 ± 0.0422	0.0653 ± 0.0495	0.0132	0.0398 ± 0.0378	0.0339 ± 0.0539	0.5633
T cells CD8	0.0842 ± 0.0443	0.0518 ± 0.0376	**0.0006**	0.0225 ± 0.032	0.0091 ± 0.0163	**0.0011**	0.0689 ± 0.0565	0.0417 ± 0.0512	**0.0023**
T cells CD4 naive	0.0123 ± 0.0219	0.008 ± 0.0169	0.1947	0.0087 ± 0.0197	0.008 ± 0.0158	0.9015	0.0005 ± 0.0128	0 ± 0	0.3185
T cells CD4 memory resting	0.0075 ± 0.0192	0.0171 ± 0.0256	**0.0141**	0.0948 ± 0.0782	0.1088 ± 0.0902	0.2897	0.2135 ± 0.0964	0.2183 ± 0.0997	0.7870
T cells CD4 memory activated	0.0144 ± 0.0214	0.0106 ± 0.0196	0.2788	0.0532 ± 0.0565	0.0405 ± 0.051	0.1414	0.0113 ± 0.0184	0.0051 ± 0.0112	**0.0105**
T cells follicular helper	0.1152 ± 0.0368	0.0928 ± 0.0304	**0.0001**	0.0764 ± 0.0434	0.0605 ± 0.0344	**0.0130**	0.0395 ± 0.0286	0.0253 ± 0.025	**0.0040**
T cells regulatory (Tregs)	0.0057 ± 0.011	0.0057 ± 0.0093	0.9907	0.0021 ± 0.0073	0.0008 ± 0.0038	0.1828	0.0296 ± 0.0341	0.03 ± 0.0249	0.9690
T cells gamma delta	0.057 ± 0.0337	0.0536 ± 0.0328	0.5443	0.0798 ± 0.0531	0.1376 ± 0.0907	**< 0.0001**	0.0011 ± 0.0066	0.0003 ± 0.0023	0.2112
NK cells resting	0.0031 ± 0.0099	0.0023 ± 0.009	0.6384	0.0118 ± 0.0185	0.0055 ± 0.0154	**0.0198**	0.0265 ± 0.0249	0.0271 ± 0.022	0.8845
NK cells activated	0.0448 ± 0.0196	0.0413 ± 0.0177	0.2811	0.0131 ± 0.0202	0.0084 ± 0.0137	0.0845	0.011 ± 0.0154	0.0068 ± 0.0108	0.0554
Monocytes	0.0091 ± 0.0173	0.0111 ± 0.018	0.4998	0.0001 ± 0.0007	0.0005 ± 0.0022	0.2281	0.0197 ± 0.0158	0.0135 ± 0.0169	0.0586
Macrophages M0	0.0684 ± 0.0347	0.0947 ± 0.0708	**0.0062**	0.0774 ± 0.0576	0.0696 ± 0.0828	0.4102	0.1279 ± 0.1083	0.2524 ± 0.1742	**< 0.0001**
Macrophages M1	0.1007 ± 0.0197	0.0972 ± 0.0242	0.3504	0.1127 ± 0.0606	0.1199 ± 0.0659	0.5825	0.0898 ± 0.0629	0.0673 ± 0.0432	**0.0115**
Macrophages M2	0.1774 ± 0.048	0.189 ± 0.0418	0.1318	0.0655 ± 0.0413	0.0809 ± 0.0506	**0.0497**	0.1726 ± 0.1199	0.1513 ± 0.0958	0.2704
Dendritic cells resting	0.0529 ± 0.0365	0.0736 ± 0.0399	**0.0017**	0.032 ± 0.0322	0.0307 ± 0.0328	0.8176	0.0053 ± 0.0143	0.0036 ± 0.0088	0.3823
Dendritic cells activated	0.0186 ± 0.0214	0.0143 ± 0.0159	0.1849	0.036 ± 0.0248	0.0193 ± 0.0262	**0.0011**	0.0192 ± 0.0341	0.0097 ± 0.0225	**0.0426**
Mast cells resting	0.0799 ± 0.0333	0.0973 ± 0.0407	**0.0067**	0.0033 ± 0.0232	0.009 ± 0.0231	0.1153	0.064 ± 0.0861	0.0364 ± 0.0322	**0.0017**
Mast cells activated	0.0008 ± 0.0034	0.0012 ± 0.0048	0.5743	0.1453 ± 0.0958	0.1359 ± 0.1424	0.6508	0 ± 0	0.0008 ± 0.0053	0.3626
Eosinophils	0.0027 ± 0.0126	0.0056 ± 0.0141	0.2142	0.0078 ± 0.0133	0.0152 ± 0.0217	**0.0203**	0.0005 ± 0.0023	0.0001 ± 0.001	0.1944
Neutrophils	0.0294 ± 0.0278	0.0307 ± 0.0231	0.7729	0.0493 ± 0.0353	0.0509 ± 0.0495	0.7458	0.0002 ± 0.0009	0.0005 ± 0.0022	0.5035

Bold values represent *P* value < 0.05.

### 3.3 PD‐L1 expression level associated with EMT status and migratory and invasive capacities in GC cell lines

**Figure 3:**
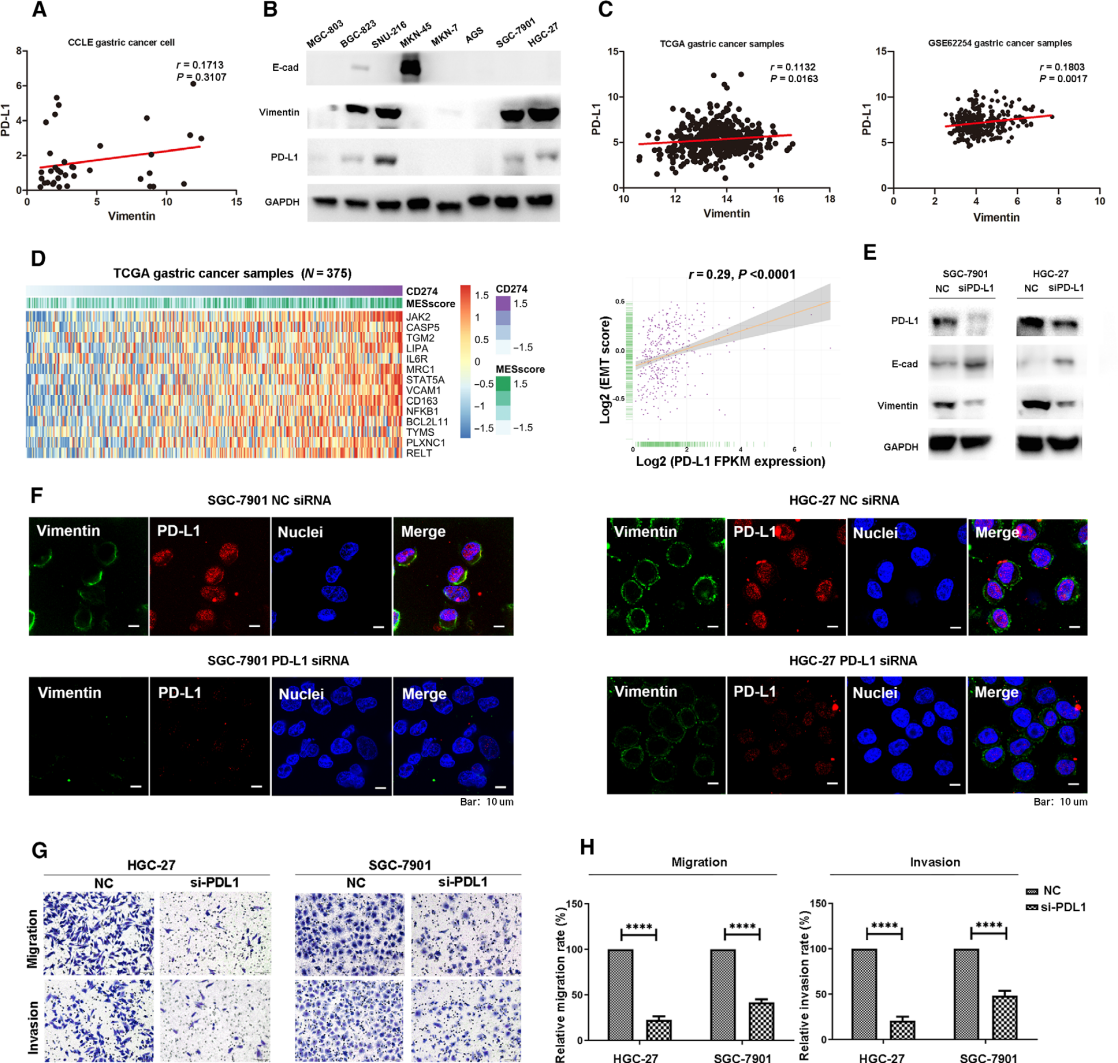
PD‐L1 promoted EMT process, migratory, and invasive capacities of GC cells. (A) Correlation between PD‐L1 and VIM mRNA expression in 37 GC cell lines of the Cancer Cell Line Encyclopedia database. (B) Western blot analysis of PD‐L1 expression and EMT markers in eight GC cell lines. (C) Correlation between PD‐L1 and VIM mRNA expression in GC patients analyzed by TCGA and http://www.ncbi.nlm.nih.gov/geo/query/acc.cgi?acc=GSE62254 databases. (D) The EMT‐related signatures enriched by high PDL1 expression in the GSVA based on TCGA GC database (left) and comparison of PD‐L1 expression with EMT score (right). (E) Western blot analysis of PD‐L1 expression and EMT markers in HGC‐27 and SGC‐7901 cells transfected with PD‐L1 siRNA or negative control siRNA (NC). (F) Immunofluorescent staining of VIM in HGC‐27 and SGC‐7901 cells transfected with PD‐L1 siRNA or negative control siRNA (NC), VIM (green), PD‐L1 (red), and nuclear stain (blue). Scale bar, 10 μm. (G) Effect of PD‐L1 knockdown on migratory and invasive capacities (magnification 20 × 10) in HGC‐27 and SGC‐7901 cells. (H) Histograms of the numbers of migrated and invaded cells. Five random fields were selected for statistical analysis. All the data are shown as mean ± SD of three independent experiments. ****P < 0.0001.

To further elucidate the role of PD‐L1 in a VIM ‐related immune cell network in GC cell lines, the expression profile data of 37 GC cell lines from the CCLE website were downloaded. As presented in Fig. 3A, positive correlation was obtained from CCLE database but did not approached statistical significance (*r* = 0.1713, *P* = 0.3107). Meanwhile, both PD‐L1 and EMT marker expressions were evaluated in 8 different GC cell lines using western blot assay. As depicted in Fig. 3B, most of the PD‐L1‐positive cell lines had mesenchymal features (MGC‐803, BGC‐823, SNU‐216, SGC‐7901, and HGC‐27), while PD‐L1‐negative cell lines displayed epithelial characteristic (MKN‐45). We further analyzed correlations in mRNA expression of VIM and PD‐L1 for patients with GC by calculating Pearson correlation coefficients. It was found that mRNA expression of VIM was positively correlated with PD‐L1 obtained from TCGA (*r* = 0.1132, *P* = 0.0163) and http://www.ncbi.nlm.nih.gov/geo/query/acc.cgi?acc=GSE62254 datasets (*r* = 0.1803, *P* = 0.0017; Fig. 3C), but not with http://www.ncbi.nlm.nih.gov/geo/query/acc.cgi?acc=GSE15459 (Fig. S1). Because of the classical hallmark of VIM in EMT process, we further calculated PD‐L1‐related EMT scores. Relative PD‐L1 expression levels were compared by EMT spectrum (Fig. 3D). The result showed that PD‐L1 mRNA expression was highly correlated with mesenchymal status (*r* = 0.29, *P* < 0.0001; Fig. 3D). The relationship between PD‐L1 and VIM expression was also validated by western blot assay and immunofluorescent staining in both of HGC‐27 and SGC‐7901 cell line after knockdown of PD‐L1 (Fig. 3E,F). The morphological changes were shown in Fig. S2. To understand the role of PD‐L1 on the migratory and invasive capacities in GC, we examined the migration and invasion of GC cell lines after knockdown of PD‐L1. As presented in Fig. 3G,H, knockdown of PD‐L1 abolished both migration and invasion ability in HGC‐27 and SGC‐7901 GC cell lines. These data indicated that the PD‐L1 expression level was associated with EMT status and migratory and invasive capacities in GC in GC cell lines.

### 3.4 Costaining of PD‐L1 and CSV in GC cell lines and tumor tissue

**Figure 4:**
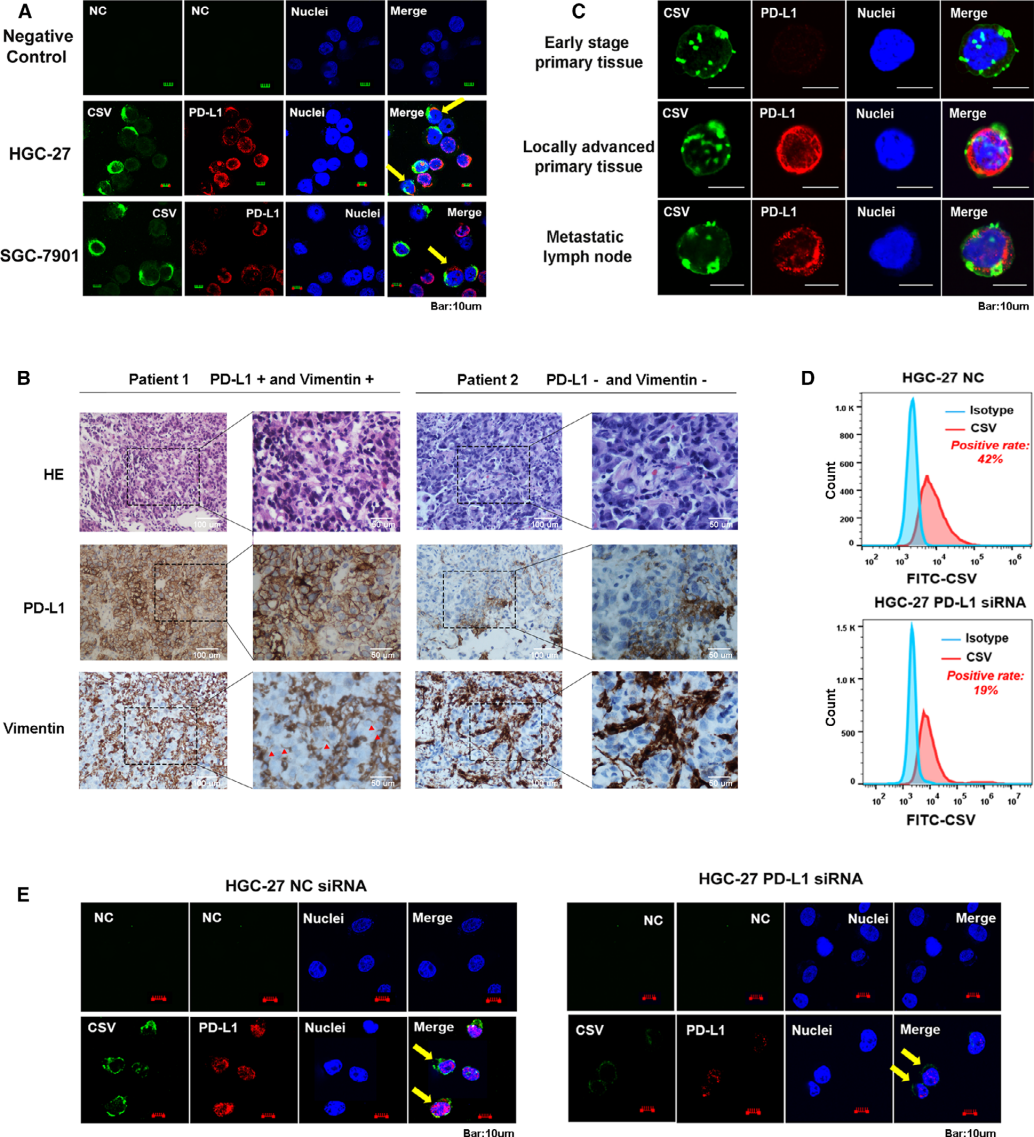
Costaining of PD‐L1 and CSV in GC cell lines and tumor tissue. (A) Immunofluorescent staining of PD‐L1 and CSV expression in both HGC‐27 and SGC‐7901 cell lines. (B) Representative images for PD‐L1 and VIM immunohistochemical staining image from two locally advanced GC cases. (C) Immunofluorescent staining of PD‐L1 after CSV+ microbeads selection from freshly resected tumor tissues obtained from three GC patients with early disease (upper), locally advanced disease (middle), and metastatic lymph node (bottom). (D) Flow cytometric evaluation of CSV expression after being transfected with PD‐L1 siRNA or NC for 48 h in HGC‐27 cell. (E) Immunofluorescent staining of PD‐L1 and CSV expression in HGC‐27 transfected with PD‐L1 siRNA or NC for 48 h. The immunofluorescent staining images are taken using confocal microscopy (magnification 10 × 10). Scale bar, 10 μm. CSV (84‐1, green), PD‐L1 (red), and nuclear stain (blue). NC, negative control, means a staining without adding the primary antibody.

Previous publications have demonstrated that VIM is a specific EMT marker and is localized on the surface of various types of cancer cells. Hence, it would be of interest to detect the costaining of PD‐L1 and CSV in GC. As shown in Fig. 4A, strong costaining of CSV and PD‐L1 could be observed in both HGC‐27 and SGC‐7901 cell lines (yellow arrows). Interestingly, it was found that two locally advanced GC patients with positive PD‐L1 expression were observed with membranous expression of VIM (CSV) but not cytoplasmic localization according to the IHC staining assay; however, neither PD‐L1 nor VIM were observed with positive expression in two other GC patients (Fig. 4B, red arrows). Likewise, single‐cell suspensions were prepared from freshly resected GC tumors or metastatic lymph node and isolated with the same protocol used for the CSV^+^CTCs analysis. As we expected, the single tumor cells from one early‐stage primary GC tissue were not detected with PD‐L1 expression; however, the single cells from locally advanced GC specimen or metastatic lymph node were observed with coexpression of CSV and PD‐L1 (Fig. 4C). Additionally, it was found that CSV expression level was significantly decreased after the knockdown of PD‐L1 based on flow cytometry and immunofluorescence data (Fig. 4D,E).

### 3.5 CSV^+^PD‐L1^+^CTCs detected in peripheral blood samples of GC patients

**Figure 5:**
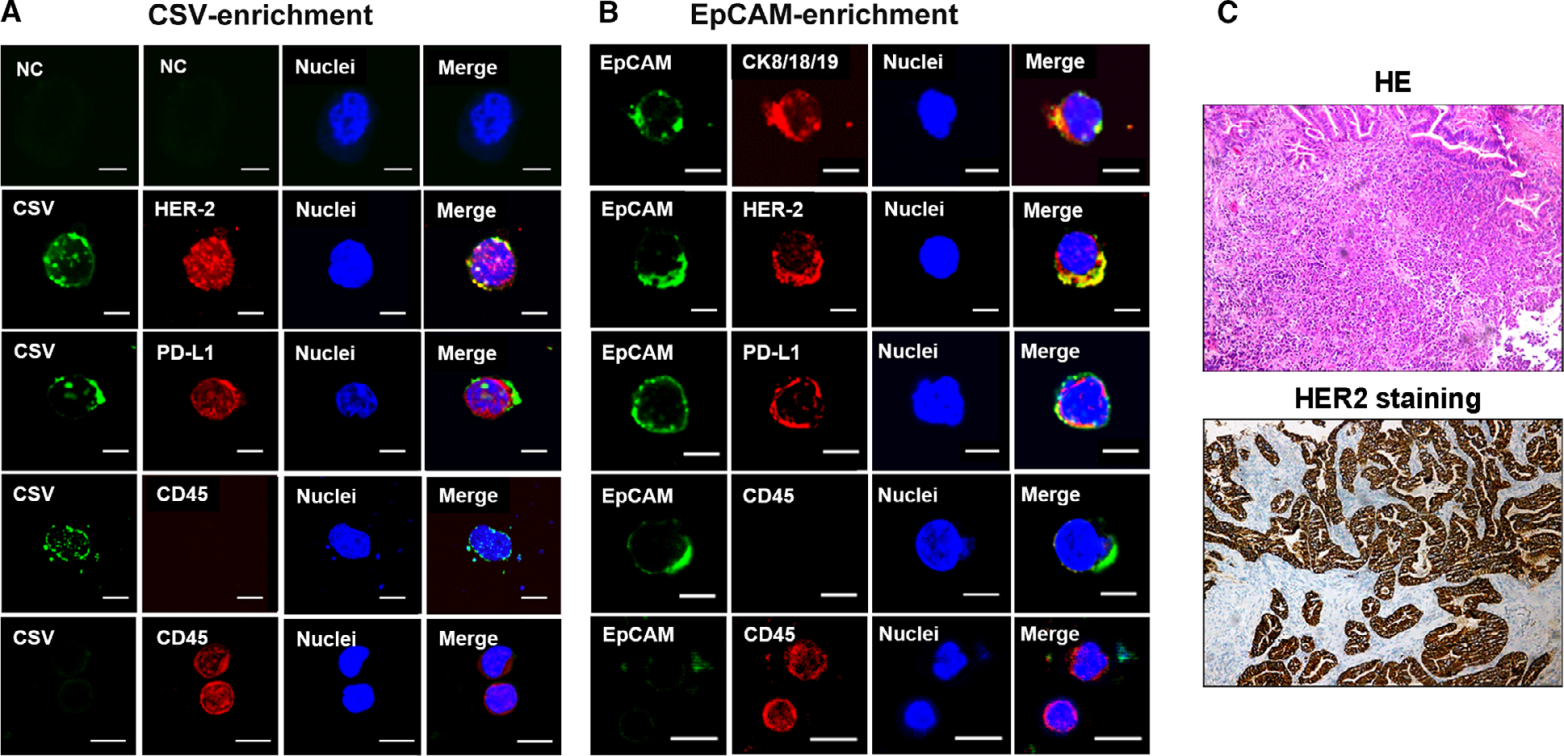
This protocol enables to capture PD‐L1^+^CTCs that express GC ‐specific marker, HER‐2. Immunofluorescent staining of CSV (84‐1, green), CD45 (red), PD‐L1 (red), EpCAM (green), and HER‐2 (red) in CTCs from an HER‐2‐positive GC patient’s blood sample captured by CSV (A) and EpCAM (B). Scale bar, 10 μm. (C) Representative images for HER‐2 immunohistochemical staining image from the same case. The original magnification is 20 × 10. NC, negative control, means a staining without adding the primary antibody.

Given that coexistence of PD‐L1 and CSV was associated with migratory and invasive capacities in GC cell lines, we hypothesized that GC cells that detached from a primary tumor and entered blood vessels would exhibit PD‐L1 expression on CTCs. It was shown that isolated CSV^+^CTCs (Fig. 5A) and EpCAM^+^CTCs (Fig. 5B) were validated by the presence of PD‐L1 expression on the membrane and in cytoplasm, however, most of EpCAM‐enriched CTCs were observed with negative expression of PD‐L1 in GC patients with early disease (Fig. S3). Furthermore, the specific tumor cell marker, HER2, was detected in both CSV and EpCAM‐enriched CTCs, which was consistent with IHC results (Fig. 5C). To further confirm that the detected CSV^+^ cells were derived from tumor tissue, analysis for whole‐genome sequencing (WGS) was performed to the CTCs isolated from one GC patient’s peripheral blood. Through the WGS of the CTC cells, we identified 7073 genes with 75 418 SNPs. Among 107 stomach specific genes, 46 intersect genes were identified in our WES dataset. Above result again proved that CSV^+^CTCs isolated from our platform were derived from GC tissues.

### 3.6 CSV^+^PD‐L1^+^CTC counts predicted disease status in GC patients

**Figure 6:**
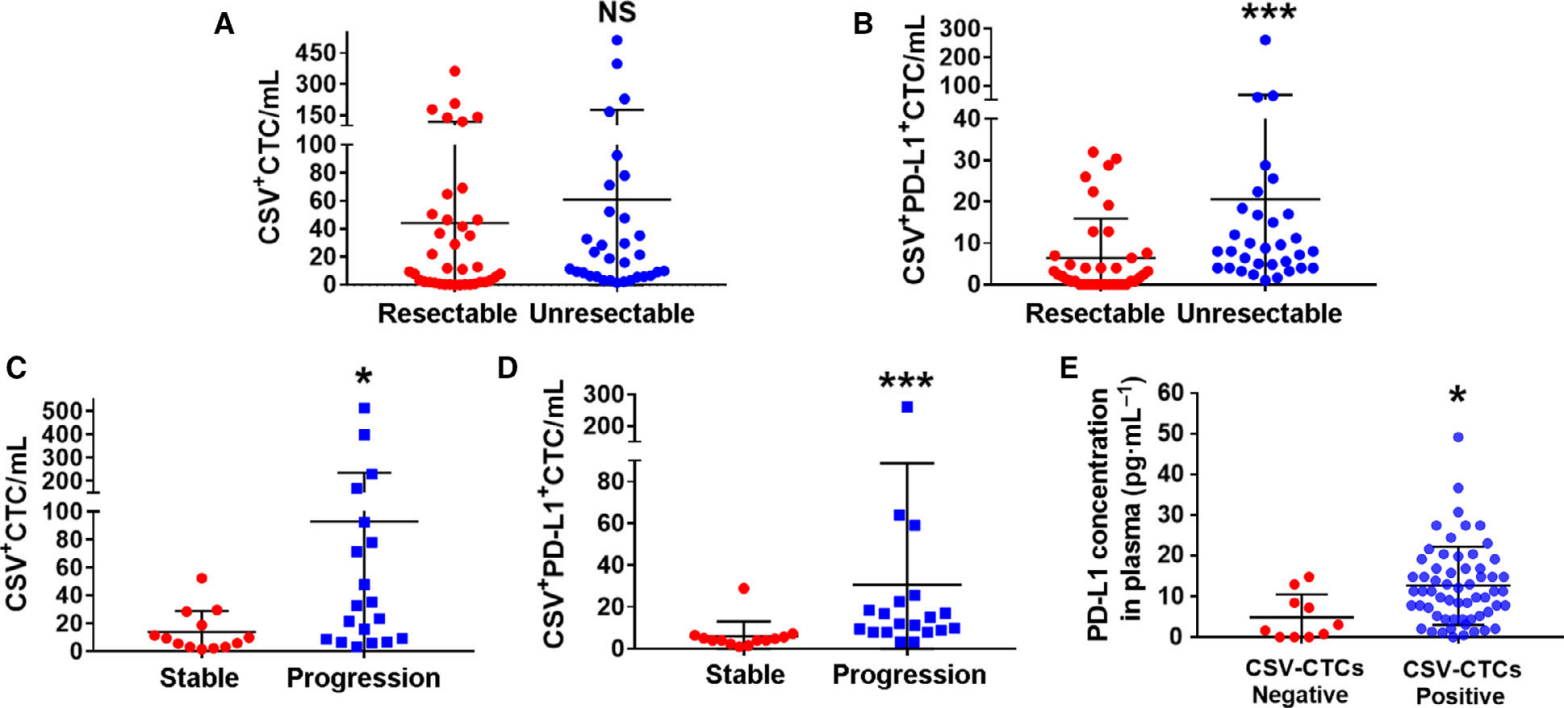
Comparison of CTCs numbers obtained from CSV isolation method grouped by patients’ disease status and therapeutic response. Comparison of total CTC counts (A) and PD‐L1^+^CTC counts (B) in patients with resectable GC or unresectable GC disease. Comparison of total CTC counts (C) and PD‐L1^+^CTC counts (D) in advanced GC patients with stable or progression disease. (E) Comparison of PD‐L1 concentration in patients with positive or negative CTCs. NS, not significant; *P < 0.05, ***P < 0.001.

Blood samples from a total of 70 patients with GC were analyzed in this study using both CSV and EpCAM microbead selection methods. The clinical characteristics of such patients are shown in Table 2. Using a predetermined cutoff value of 1 CTC per mL of blood sample (equal to 5 CTCs/7.5 mL based on the CellSearch method), CTCs were detectable in 60 of the 70 (86%) GC patient samples in our study, ranging from 0 to 512 mL^−1^; CSV^+^PD‐L1^+^CTCs were found in 50 of the 70 (71%) GC patient samples, ranging from 0 to 261 mL^−1^ (Fig. 6A,B, Table 3). The enrolled patients were divided into resectable and unresectable groups according to disease status at the time of blood collection. In comparison with total CTC counts, CSV^+^ PD‐L1^+^CTCs showed a significant difference in distinguishing resectable and unresectable populations (Fig. 6B, 2 vs. 8 mL^−1^, *P* < 0.001). Further, we classified unresectable patients with measurable lesions into (a) stable or responding disease, or (b) progressive disease based on RECIST guidelines. PD‐L1^+^CTC counts (8 mL^−1^) were selected as optimal cutoff value determined by ROC curves with best sensitivity and specificity (data not shown). Of these 32 patients with advanced GC, 13 (41%) exhibited a radiographic response or stable disease, and 19 (59%) had progressive disease (Table 4). We observed a significant difference in response to therapy by total CSV^+^CTC counts (Fig. 6C, *P* < 0.05) and CSV^+^PD‐L1^+^CTC counts (Fig. 6D, *P* < 0.01). Meanwhile, we detected PD‐L1 concentration in GC patients’ plasma using Luminex assay. As shown in Fig. 6E, PD‐L1 levels were obviously higher in CSV^+^CTCs patients compared with the negative group (4.9 pg·mL^−1^ ± 1.78 vs. 12.7 pg·mL^−1^ ± 1.24, *P* = 0.016)). Thus, CSV^+^PD‐L1^+^CTC enumerations were potential to be better predictive markers for evaluating disease status and therapeutic responses in patients with GC.

**Table 2:** Clinical characteristics of GC patients in this study (*N* = 70).

Clinical characteristic	Number (%)
Sex	
Male	51 (73)
Female	19 (27)
Age, years old	
< 63	32 (46)
≥ 63	38 (54)
Disease status	
Resectable	38 (54)
Unresectable	32 (46)
Treatment strategies	
Systemic chemotherapy	53 (76)
Supportive treatment	17 (24)

**Table 3:** Association of CTC counts with clinical features in GC patients (*N* = 70).

Characteristic	CSV^+^CTCs			PD‐L1^+^CTCs		
	Negative	Positive	*P* value	Negative	Positive	*P* value
Sex						
Male	7 (10%)	44 (63%)	0.548	14 (20%)	37 (52%)	0.475
Female	3 (4%)	16 (23%)		6 (9%)	13 (19%)	
Age, years old						
< 63	5 (7%)	27 (39%)	0.516	13 (19%)	19 (27%)	0.037
≥ 63	5 (7%)	33 (47%)		7 (10%)	31 (44%)	
Disease status						
Resectable	9 (13%)	29 (41%)	0.003	18 (26%)	20 (28%)	< 0.001
Unresectable	0 (0%)	32 (46%)		0 (0%)	32 (46%)	
TNM						
Ⅰ‐Ⅲ	7 (10%)	28 (40%)	0.153	14 (20%)	21 (30%)	0.031
Ⅳ	3 (4%)	32 (46%)		6 (9%)	29 (41%)	
Grade						
High/intermediate	3 (4%)	17 (24%)	0.553	6 (9%)	14 (20%)	0.563
Low	4 (6%)	28 (40%)		9 (12%)	23 (33%)	
NA	18 (26%)			18 (26%)		
Metastasis						
Yes	1 (1%)	25 (36%)	0.530	2 (3%)	24 (34%)	0.002
No	9 (13%)	35 (50%)		18 (26%)	26 (37%)	
Treatment strategies						
Systemic chemotherapy	8 (12%)	45 (64%)	0.094	16 (23%)	37 (53%)	0.006
Supportive treatment	0 (0%)	17 (24%)		0 (0%)	17 (24%)	

**Table 4:** Association of PD‐L1^+^CTC counts with clinical features in unresectable GC patients (*N* = 32).

Characteristic	PD‐L1^+^CTCs		*P* value
	< 8 mL^−1^ (%)	≥ 8 mL^−1^ (%)	
PS score			
0–1	12 (37)	14 (44)	0.460
> 2	2 (6)	4 (13)	
Lines of therapies			
First/second line	10 (31)	5 (15)	0.039
Supportive treatment	5 (16)	12 (38)	
Therapeutic response			
PR/SD	12 (38)	1 (3)	< 0.001
PD	2 (6)	17 (53)	
Liver metastasis			
Yes	1 (3)	4 (12)	0.255
No	13 (41)	14 (44)	

### 3.7 Correlation between CSV^+^PD‐L1^+^CTC counts and survival in GC patients

**Figure 7:**
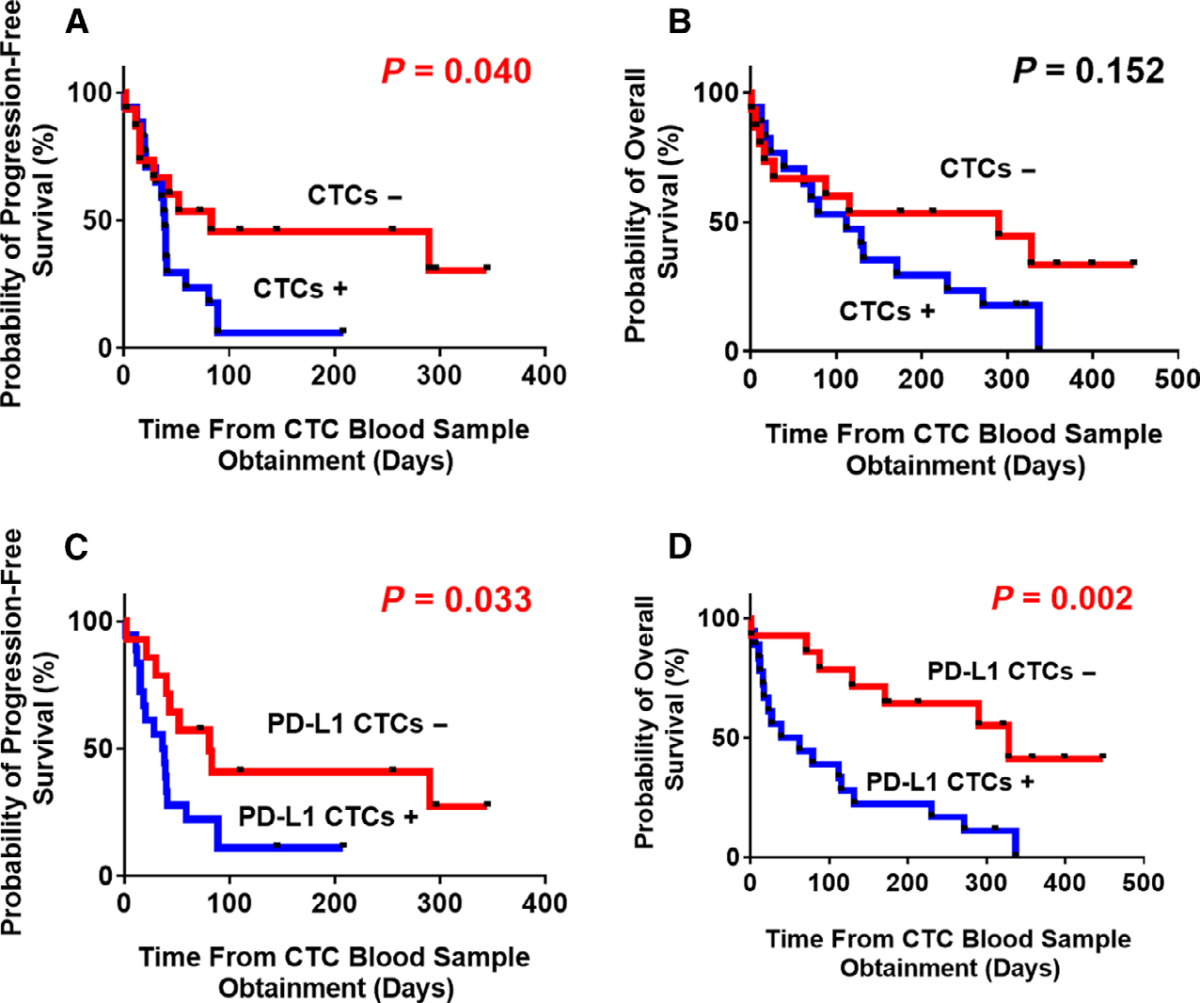
Kaplan–Meier analysis of PFS for advanced GC patients grouped by (A) total CTC counts or (C) PD‐L1^+^CTC counts at baseline of blood draw; and OS according to (B) total CTC counts or (D) PD‐L1^+^CTC counts at baseline.

We further assessed CSV^+^PD‐L1^+^CTC enumerations for their prognostic significance in patients with advanced GC. From our observations, we defined a median value of 8 mL^−1^ as the optimal cutoff for PD‐L1^+^CTCs with respect to prognostic prediction using ROC curves. At a median follow‐up of 12.9 months, total CTC counts yielded a HR of 2.364 for PFS (95% CI: 1.038–5.381; *P* = 0.040; Fig. 7A) and 1.817 for OS (95% CI: 0.8025–4.114; *P* = 0.152; Fig. 7B). However, we observed that patients with PD‐L1 overexpression in the CSV^+^CTC population at the baseline of the blood collection showed decreased PFS (HR: 2.437; 95% CI: 1.074–5.529; *P* = 0.033) and worse OS (HR: 3.762; 95% CI: 1.629–8.691; *P* = 0.002) compared to patients with lower numbers of PD‐L1^+^CTCs (Fig. 7C,D).

**Figure 8:**
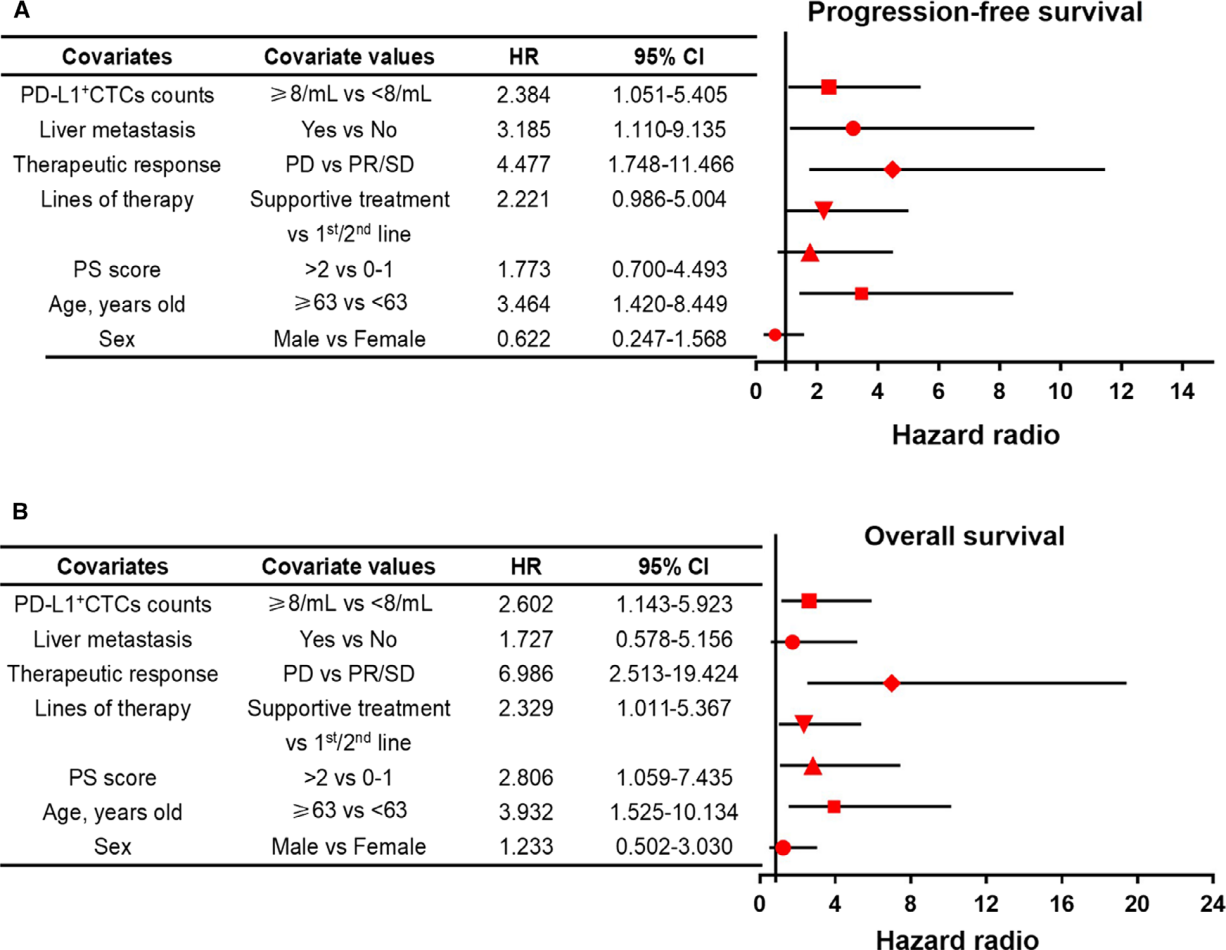
Forest plots of univariate Cox models for (A) PFS and (B) OS for advanced GC patients.

Univariate analysis also revealed a significantly higher risk of disease progression in patients with advanced GC and a CSV^+^CTC fraction showing PD‐L1 overexpression compared with the PD‐L1 low expression group. A multivariate Cox regression model confirmed that a performance status score > 2, old age, and disease progression were independently associated with worse PFS and OS (*P* < 0.05). Although patients with PD‐L1 overexpression in CSV^+^CTCs appeared to have worse outcomes, this was not an independent prognostic variable for the advanced GC cohort. Forest plots of univariate Cox‐regression hazard models are presented in Fig. 8A,B. However, these require further evaluation in a prospective study with large samples and a long follow‐up time.

## 4 Discussion

Although advancements in the treatment of advanced GC have led to improvements in making a prognosis, this has only benefited a few subpopulations of patients with GC. Hence, new prognostic markers that can select patients who have a higher risk of relapse and that potentially predict survival are still needed. CTCs have recently received attention as probes that guide the monitoring of therapeutic efficacy in patients with various types of cancers (Hamilton and Rath, 2018; Li *et al.*, 2018). Considering the role of the EMT process in drug resistance and tumor metastasis, EMT‐CTCs may be key determinants in the prediction of a prognosis in patients with advanced cancer (Mitra *et al.*, 2015; Satelli *et al.*, 2015a; Satelli *et al.*, 2015b). Many researchers have investigated the enrichment of EMT‐CTCs in breast, colorectal and prostate cancers and their associations with aggressive phenotypes (Satelli *et al.*, 2017; Satelli *et al.*, 2015b). Recently, we successfully demonstrated the detection of CTCs from peripheral blood samples of patients with advanced GC with high sensitivity based on an EMT‐CTC specific selection marker, CSV (unpublished data). However, CTCs are a heterogeneous cell population. For example, some cells can evade the host immune system to induce rapid cancer progression. These limitations prompted us to look for precise markers or a marker panel of CSV^+^ CTCs for potential opportunities in advanced GC.

Nowadays, investigations of PD‐L1 have gained momentum due to its importance in cancer progression and metastasis (Alsuliman *et al.*, 2015). Blocking the PD‐1/PD‐L1 checkpoint pathway is now an attractive medical approach to activate T lymphocytes and enhance antitumor immunity (Zou *et al.*, 2016). In this present study, PD‐L1 mRNA expression was highly correlated with EMT status and migratory and invasive capacities based on published gene expression datasets and *in vitro* data. Meanwhile, there is a growing trend in analysis of the PD‐L1 expression level in CTCs in this field. A recent study has indicated that nuclear PD‐L1 expression in CTC fractions can predict the prognosis for colorectal and prostate cancers (Satelli *et al.*, 2016a). The utility of PD‐L1 + CTCs detection is feasible and provides important prognostic information in head and neck squamous cell carcinoma patients (Strati *et al.*, 2017). However, the evaluation of PD‐L1 protein status in GC‐CTCs is not elucidated at present.

Besides PD‐L1, VIM is another marker correlated with immune status in patients with GC based on bioinformatics data in this study. Previous studies of VIM focused on its essential role in cellular network junctions as a classical EMT marker. To further explore VIM ‐related biological functions in GC, we performed functional analysis of VIM using the Hallmark Gene Sets. Our results for the first time suggested that the higher expression of VIM was obviously correlated with the immune‐related pathway, especially associated with CD8^+^ T‐cell inhibition in patients with GC. Moreover, VIM was upregulated in diffuse GC tissues and associated with a poor prognosis. Hence, detection of both VIM and PD‐L1 expression on CTC cells may potentially be more clinically relevant. However, intracellular expression of VIM in most of the immune cells limits its utility as a CTC marker. As we mentioned above, cytoplasmic VIM translocated to the tumor CSV has been reported by several previous publications. Monoclonal antibody 84–1, which is specific to CSV, has been used to capture CTCs from various types of cancers successfully with high sensitivity and specificity. Therefore, real‐time sampling of patients with CTCs during a therapy period would provide information on mechanisms of tumor escape based on PD‐L1^+^CSV^+^CTC enrichment method.

Building on our pilot study, for the first time, we revealed the coexpression of CSV and PD‐L1 in GC cell lines, single‐cell suspensions isolated from tumor specimens, tumor tissue sections and CTCs derived from the peripheral blood samples of GC patients. According to our present study, CSV^+^PD‐L1^+^CTCs in patients were associated with a late disease status and poor response. Furthermore, we defined a cutoff of <eight or ≥eight CSV^+^PD‐L1^+^CTCs as an optimal threshold with respect to a therapeutic response and prognosis using ROC curves that showed improved sensitivity and specificity. From our observations, patients with PD‐L1 overexpression in CSV^+^CTC cell populations could not obtain a clinical benefit and this predicted a poor prognosis. Thus, the persistence of CSV^+^PD‐L1^+^CTCs may represent a mechanism of therapy escape. Together, these results suggest that CSV and PD‐L1 are potential biomarkers in the selection of GC patients with a high risk of metastasis and a poor prognosis. To our knowledge, this is the first study to provide clinically relevant data to confirm CTCs detection using both PD‐L1 and CSV‐specific markers in patients with GC.

Limitation of this pilot study has to be considered. Firstly, this was a pilot study and only one time point of blood sample was considered for CTCs evaluation. A prospective clinical trial with larger sample size and different time point of blood collections would be essential to understanding how PD‐L1^+^CTCs could be used to predict relapse and prognosis. Secondly, the molecular mechanism of how PD‐L1 induces the up‐regulation of intracellular VIM expression to the cell surface in the process of metastasis in GC has not been explored. Studies indicated that VIM translocated to tumor cell surface was phosphorylation‐dependent process (Satelli *et al.*, 2014). Moreover, exogenously supplemented VIM could bind to the surface of the cancer cells and activated Wnt signaling pathway by enhancing phosphorylation of β‐catenin with accumulation in the nucleus; circulating VIM secreted into the blood bound to the cell surface would promote cellular invasive properties *in vitro* (Satelli *et al.*, 2016b). Hence, the above scenarios require further *in vitro* and *in vivo* investigations in GC model.

## 5 Conclusion

In conclusion, we report that the detection of PD‐L1^+^CTCs in peripheral blood using a CSV method predicts a therapeutic response and prognosis in patients with GC. The use of CTC‐based models in GC risk assessment may improve the standard of staging criteria and support the incorporation of PD‐L1 expression for the detection of CTCs in such models. Our results provide an important framework for further multicenter prospective studies in this field.

## Conflict of interest

The authors declare no conflict of interest.

## Author contributions

Conception and design: MS and HL. Data acquisition, analysis, and interpretation: ML, RW, XS, YL, ZW, JY, XK, TZ, XJ, GW, FW, and GW. Drafting of the manuscript or revising it critically for important intellectual content: ML, RW, and HL. IHC staining and analysis: QZ and WL. Administrative, technical, or material support: ML, YL, ZW, JY, and SL.

## Supporting information


**Fig. S1.** Correlation between PD‐L1 and VIM mRNA expression in gastric cancer patients analyzed by http://www.ncbi.nlm.nih.gov/geo/query/acc.cgi?acc=GSE15459 databases.

**Fig. S2.** Photos were taken at 10 × 20 magnification after being transfected with PD‐L1 siRNA or NC for 48 h in SGC‐7901 gastric cancer cell line.

**Fig. S3.** Immunofluorescent staining of CK8/18/19 (red), CD45 (red), PD‐L1 (red), EpCAM (green) in CTCs from a GC patient’s blood sample captured by EpCAM. Scale bar, 10μm. The original magnification is 10 × 20. NC, negative control, means a staining without adding the primary antibody.

**Table S1.** Antibody resources table.
