# Supplementary figures and images for: Prognostic significance of PD‐L1 expression on cell‐surface vimentin‐positive circulating tumor cells in gastric cancer patients
Source: Mol Oncol. 2020 Feb 28;14(4):865–81. doi: 10.1002/1878-0261.12643 (PMC7138401; doi:10.1002/1878-0261.12643)

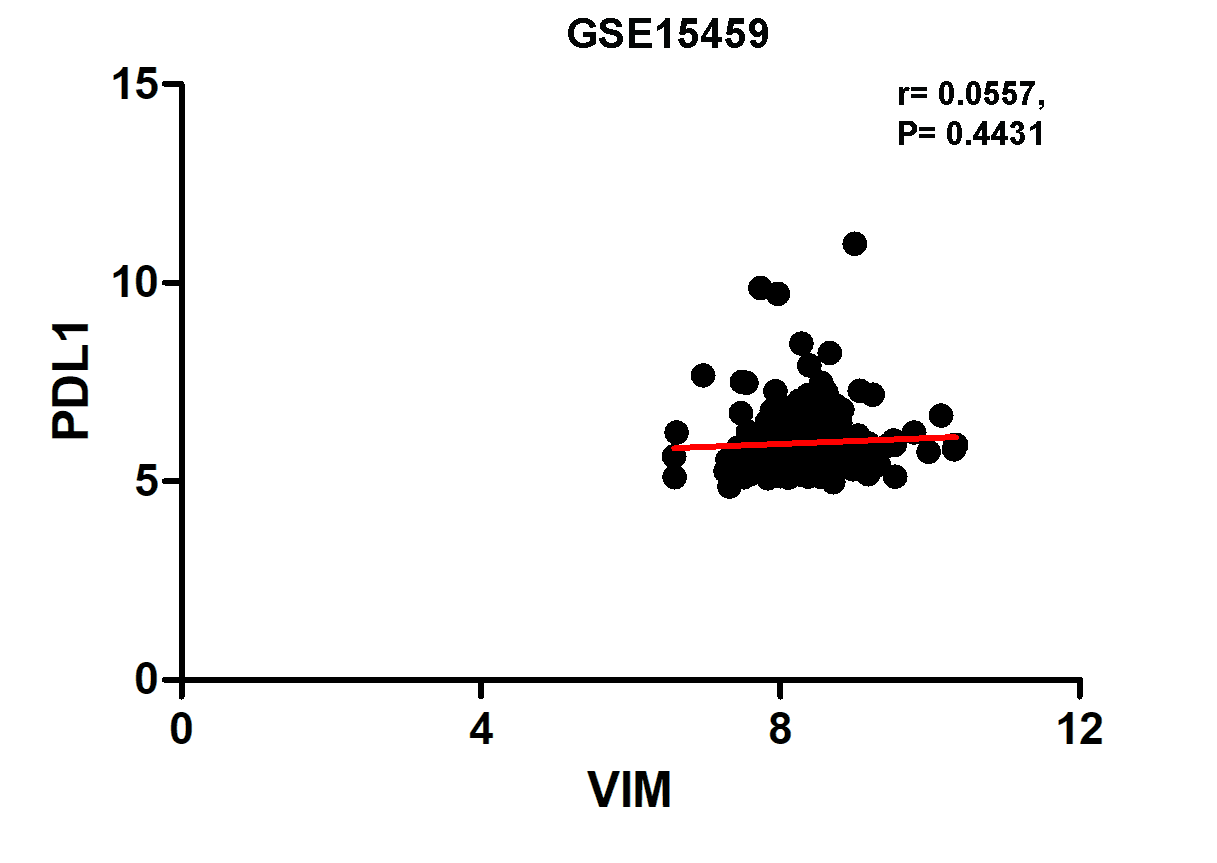

Supplement: Supplementary file 1 — Fig. S1. Correlation between PD‐L1 and VIM mRNA expression in gastric cancer patients analyzed by http://www.ncbi.nlm.nih.gov/geo/query/acc.cgi?acc=GSE15459 databases. [file MOL2-14-865-s001.tif]

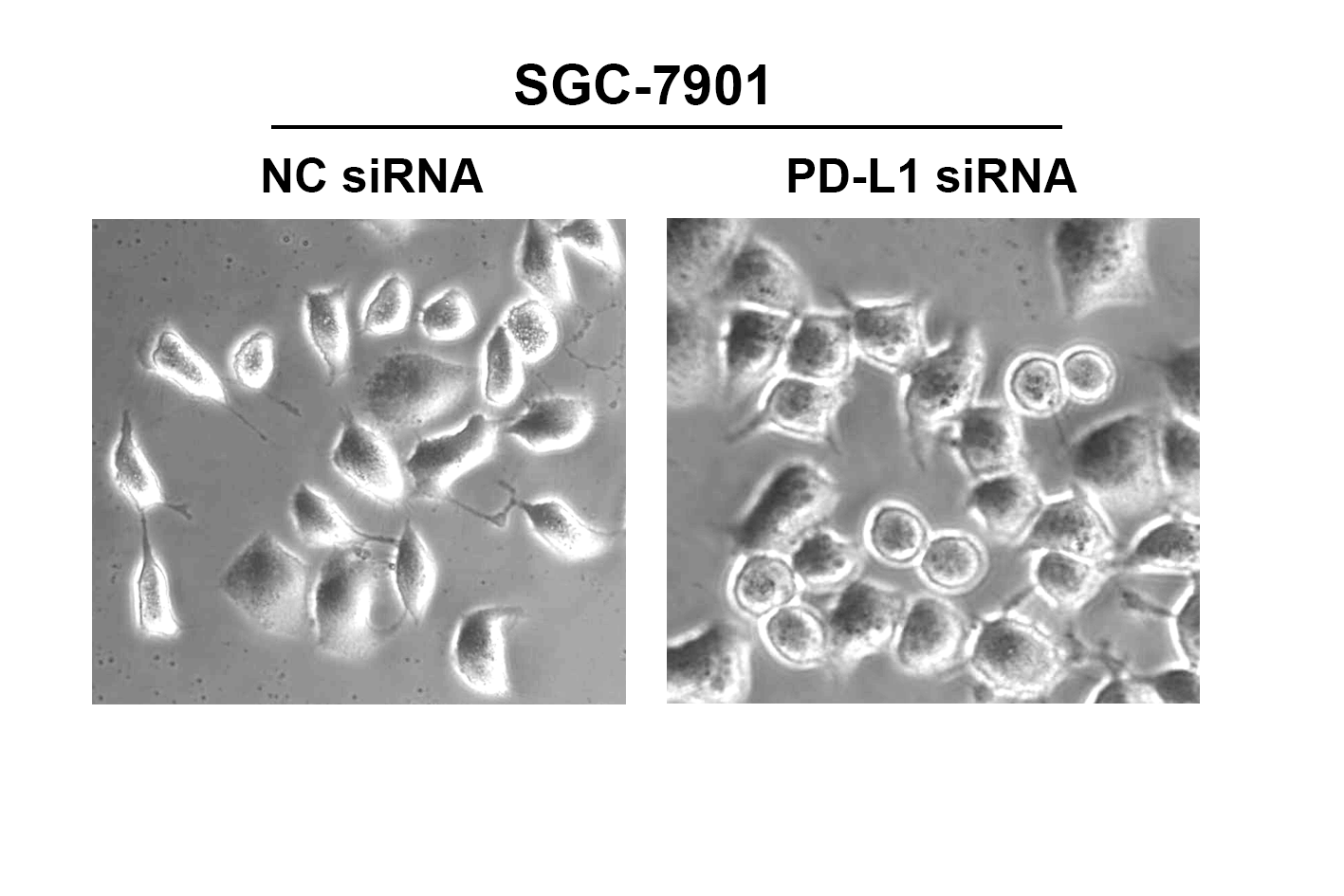

Supplement: Supplementary file 2 — Fig. S2. Photos were taken at 10 × 20 magnification after being transfected with PD‐L1 siRNA or NC for 48 h in SGC‐7901 gastric cancer cell line. [file MOL2-14-865-s002.tif]

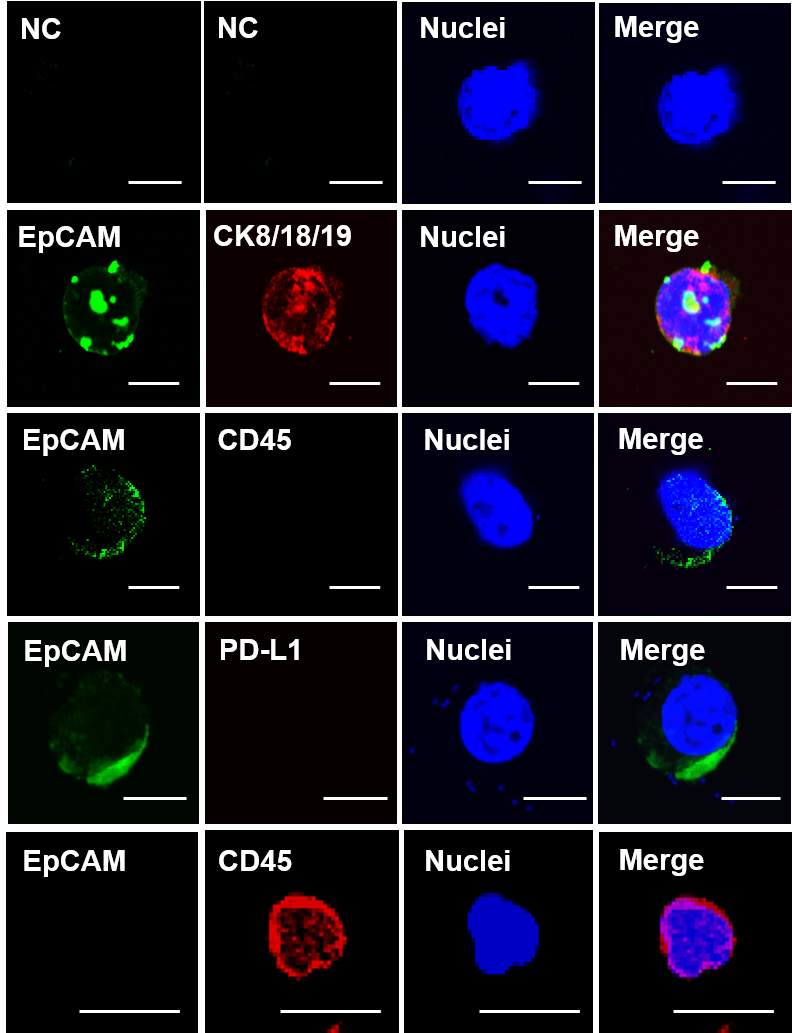

Supplement: Supplementary file 3 — Fig. S3. Immunofluorescent staining of CK8/18/19 (red), CD45 (red), PD‐L1 (red), EpCAM (green) in CTCs from a GC patient’s blood sample captured by EpCAM. Scale bar, 10μm. The original magnification is 10 × 20. NC, negative control, means a staining without adding the primary antibody. [file MOL2-14-865-s003.tif]
