# Supplementary material for: Prognostic significance of PD‐L1 expression on cell‐surface vimentin‐positive circulating tumor cells in gastric cancer patients
Source: Mol Oncol. 2020 Feb 28;14(4):865–81. doi: 10.1002/1878-0261.12643 (PMC7138401; doi:10.1002/1878-0261.12643)
Supplement: Supplementary file 4 — Table S1. Antibody resources table. [file MOL2-14-865-s004.docx]

| **Antibodies** | **Clone** | **Source** | **Dilution** |
| --- | --- | --- | --- |
| Anti-Mouse CSV | 84-1 | Abnova | 1:100 |
| Anti-Rabbit EpCAM | D4K8R | CST | 1:200 |
| Anti-Mouse EpCAM | VU1D9 | CST | 1:500 |
| Anti-Rabbit CD45 | D9M8I | CST | 1:500 |
| Anti-Rabbit HER-2 | 29D8 | CST | 1:200 |
| Anti-Rabbit E-cadherin | 24E10 | CST | 1:1000 |
| Anti-Rabbit Vimentin (WB) | D21H3 | CST | 1:1000 |
| Anti-Rabbit GAPDH | D16H11 | CST | 1:1000 |
| Anti-Rabbit PD-L1 (IF) | D8T4X | CST | 1:200 |
| Anti-Rabbit Vimentin (IHC) | MX034 | Maixin | NA |
| Anti-Rabbit PD-L1 (IHC) | 22C3 | Dako | 1:50 |
| Anti-Rabbit PD-L1 (WB) | E1L3N | CST | 1:1000 |
| Secondary Anti-mouse Alexa Fluor-488 | F(ab')2 | CST | 1:500 |
| Secondary Anti-rabbit Alexa Fluor-555 | F(ab')2 | CST | 1:500 |
| DAPI | NA | Solarbio | 1:400 |

**Antibody resources table**
